# Automation of photochemical kinetic determinations without irradiation of the detection cell

**DOI:** 10.1155/S1463924691000214

**Published:** 1991

**Authors:** M. T. Tena, M. D. Luque de Castro, M. Valcarcel

**Affiliations:** Department of Analytical Chemistry Faculty of Sciences University of Córdoba Córdoba 14004 Spain

## Abstract

This paper reports on the development of an open-closed configuration for continuous kinetic methods based on photochemical reactions; the configuration removes the need to use special flow cells for simultaneous irradiation and detection. The sample plug was recirculated through a circuit including the detector and a coil irradiated with an ultra-violet source. A multipeak recording per injected sample was obtained which allowed signal increments between peaks (whether successive or not) to be measured. The configuration was applied to the fluorimetric determination of thioridazine. The log-log calibration curve obtained was linear in the range over 0.1-20.0 μg/ml of the analyte. The sampling frequencies achieved were between 24 and 13 h^-1^, depending on the peaks which were selected for measurement. The method was applied to the determination of the analyte in pharmaceuticals.


Journal of Automatic Chemistry, Vol. 13, No. 3 (May–June 1991), pp. 111–113

### Automation of photochemical kinetic determinations without irradiation of the detection cell

**M. T. Tena, M. D. Luque de Castro and M. Valcarcel**
*Department of Analytical Chemistry, Faculty of Sciences, University of Córdoba, 14004 Córdoba, Spain*

*This paper reports on the development of an open–closed configuration for continuous kinetic methods based on photochemical reactions; the configuration removes the need to use special flow-cells for simultaneous irradiation and detection. The sample plug was recirculated through a circuit including the detector and a coil irradiated with an ultra-violet source. A multipeak recording per injected sample was obtained which allowed signal increments between peaks (whether successive or not) to be measured. The configuration was applied to the fluorimetric determination of thioridazine. The log-log calibration curve obtained was linear in the range over 0·1–20·0 μg/ml of the analyte. The sampling frequencies achieved were between 24 and 13 $h^{-1}$, depending on the peaks which were selected for measurement. The method was applied to the determination of the analyte in pharmaceuticals.*

Photosensitizing compounds can be easily assayed qualitatively and quantitatively; thus non-photosensitizing compounds can be determined indirectly. A number of methods have been developed using photochemical reactions, particularly in pharmaceutical analysis [1].

Flow Injection Analysis (FIA) provides photochemical determinations with an additional, advantage: a high reproducibility in the working conditions used, which, among other things, allows irradiation to be applied in a very precise manner by controlling the exposure time (residence time of the sample in the irradiated reactor), and the relative position of the light source and the sample. The FIA methods developed require very simple manifolds because it is unnecessary to use any reagent. Individual [2] and simultaneous [3] determinations of phenothiazine compounds, of oxalate in urine [4], and of organic phosphorus in natural waters [5] are good examples of these methods; they all involve making fixed-time measurements after irradiation for a preset time. Only in one of these methods was the analyte (oxalate) irradiated in the flow-cell (an electrochemical cell), in order to monitor the course of the reaction. These reaction-rate measurements provide the method in question with the increased selectivity of kinetic methods [6]. However, only special flow-cells and compartments can be irradiated. In order to solve this problem, which is particularly serious in molecular optical detectors, an FIA manifold was designed, based on the principles of open–closed systems [7–9], which have excellent performance in the determination of thioridazine. The photoactivity of this tranquillizer allowed the development of selective, fast fluorimetric methods for its determination.

### Experimental

### *Instrument and apparatus*

A Kontron SFM25 spectrofluorimeter, with a flow-cell of 18 μl inner volume, and a Perkin-Elmer LS-1 fluorimeter provided with another flow-cell of 4 μl inner volume, both equipped with Knauer x-t recorders, were used to obtain spectra and perform fixed-time wavelength measurements, respectively. An Eppendorf EVA peristaltic pump, two Rheodyne 5041 injection valves (one of them acting as a selecting valve) and a Uvatom-70 UV lamp were also used.

### *Reagents*

Aqueous stock solutions containing 1·00 g/l thioridazine (Aldrich), $5 \times 10^{-3}$ M potassium peroxodisulphate, and 0·5M sodium citrate/citric acid were prepared. From these, more dilute solutions were prepared as necessary.

### *Manifold and data processing*

The manifold used is shown in figure 1. The sample was injected into a 0·1M citrate buffer of pH 3·0 used as carrier and containing $1 \times 10^{-4}$M potassium peroxodisulphate for transport to the detector. Then, the selecting valve (SV) was switched to its closed position and the sample plug passed through the coil under irradiation and then through the flow-cell again. Successive passage of the plug through the irradiated coil and the flow-cell gave rise to a multipeak recording (see figure 2), with as many peaks as times the sample was circulated through the closed circuit. After the recording was obtained, SV was switched to its open position and the sample was sent to waste, the circuit being made ready for insertion of a

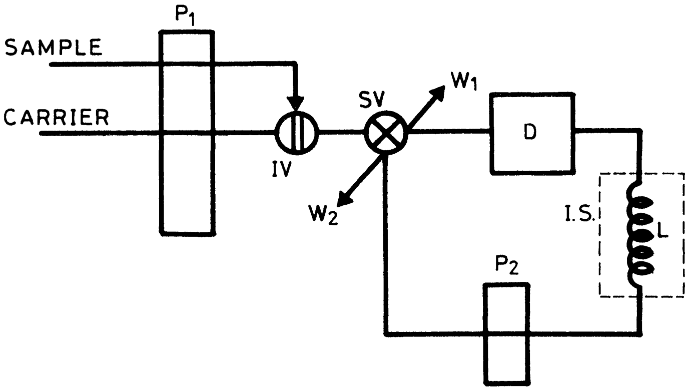

*Figure 1. Open–closed manifold with irradiation of the coil for obtaining of kinetic measurements. P denotes the peristaltic pump, IV the injection valve, W the waste, D the detector, IS the irradiation system, and, L, the reactor.*

0142–0453/91 $3.00 © 1991 Taylor & Francis Ltd.

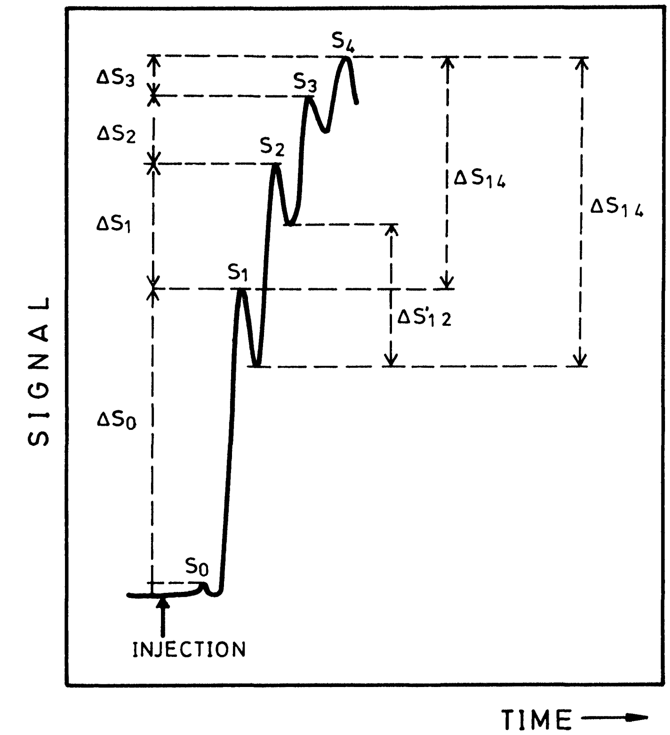

*Figure 2. Different analytical measurements that can be derived from the multipeak recording. Signal increment between peak maximum (whether successive or not), between minima and maxima or between minima.*

new sample. The relative positions of the detector and irradiated coil were crucial for obtaining a first peak which was as low as possible.

As shown in figure 2, various types of kinetic measurements can be performed on the multipeak recording after the blank signal (a recording obtained with no irradiation of the sample) was subtracted. $\Delta S_o$ corresponds to the fluorescence intensity increment between the first peak (before the sample had passed through the irradiated reactor) and the second. Other measurements were made between maxima, a maximum and a minimum, or between minima, whether successive or not. The sensitivity of the calibration curve can be improved by summing several $\Delta S_i$ values. Although a recorder was used to study the behaviour of the system, the data were collected by a microcomputer and were processed with a simple program, which save the logarithm of the fluorescence intensity increment between two preselected peaks after the corresponding blank was subtracted (log $[(S_n - S_{n-1})_S - (S_nS_{n-1})_B)]$. This value was compared with the data from the calibration curve, and the result (expressed in μg/ml) was either printed out or displayed on the monitor.

### Results and discussion

*Basis of the method*

Thioridazine exhibits a very weak native fluorescence at 455 nm on excitation at 325 nm. In the presence of a suitable UV-visible source, it forms an oxidized derivative with a more rigid structure and a very intense fluorescence ($\lambda_{ex}$ = 340 nm, $\lambda_{em}$ = 390 nm).

*Preliminary studies*

Preliminary batch assays were conducted in order to determine the best irradiation wavelength and the influence of the exposure time. A conventional cell was used to position the sample in the spectrofluorimeter is light-path in order to pinpoint the irradiation wavelength. The highest fluorescence intensity was obtained by irradiating at 260 nm, so a Uvatom-70 source emitting at this wavelength was used for irradiation. The signal increased dramatically with an increase in the exposure time, the relative fluorescence intensity was 0·8, 3·2 and 6·5 for irradiation times of 1, 5 and 10 min, respectively; thus, the sensitivity can be improved at the expense of sampling frequency.

*Optimization of variables*

Chemical and interdependent variables (for example FIA variables) were optimized by the univariate method and modified simplex method, respectively.

*Chemical variables*

Despite the fact that the literature generally considers a pH close to 8 to be optimal [10,11], pH 3·0 provided a higher signal and a smaller blank. Different buffer solutions (citrate, chloracetate and phosphate) at different concentrations were assayed. The best results were obtained by using a 0·1 M sodium citrate/citric acid buffer.

The presence of an oxidant, such as potassium peroxodisulphate, favours the oxidation of the analyte [10]. The multipeak recording obtained by using irradiation increased with increasing concentration of the oxidant; however, the multipeak recording obtained in the absence of irradiation was somewhat higher. A compromise between both extremes was made by selecting a concentration of $1 \times 10^{-4}$ M.

Application of the modified simplex method for optimization of the injected volume ($V_i$), flow-rate in the closed circuit ($q$), and length of the reactor $L$, showed 530 μl, 1·61 ml/min and 100 cm (0·5 mm ID), respectively, to providing the highest response function (maximum difference between successive peaks after subtraction of the blank signal).

The influence of the flow-rate was studied in several experiments with the univariate method. Reducing the flow-rate by 50% (from 1·6 to 0·8 ml/min) resulted in an increase in the height of the first peak of about 80%. Under these working conditions, the second and successive peaks decreased gradually as a result of the prevalent effect of dispersion on the reaction evolution. Thus, the flow-rate can be manipulated to obtain increased sensitivity if $\Delta S_o$ is used as analytical signal (see figure 2).

The switching time ($ST$), i.e. the interval between injection of the sample and the instant at which the selecting valve was switched from its open to its closed position, was a key variable because it determined whether or not the overall plug was trapped in the closed circuit. An $ST$ of 45 s was chosen as optimum.

*Table 1. Features of the method.*

| Analytical signal* | Slope | Intercept | Regression coefficient | Determination range, μg/ml | Sampling frequency, $h^{-1}$ |
|---|---|---|---|---|---|
| $\Delta S_0$ | 0·737 | 0·305 | 0·9988 | 0·1–20·0 | 24 |
| $\Delta S_1$ | 0·955 | $-3{\cdot}582 \times 10^{-2}$ | 0·9997 | 0·1– 5.0 | 17 |
| $\Delta S_2$ | 0·754 | −0·226 | 0·9999 | 0·5–20·0 | 13 |

* See figure 2.

*Features of the calibration curves*

A series of standards with concentrations between 0·05 and 30·0 μg/ml were injected into the system; the results obtained were processed in different ways to establish signal-concentration relationships. A log-log calibration curve per measurement parameter was obtained. Table 1 shows the parameters used to construct the curves, the linear range and sensitivity (slope), which depend on the parameter selected. The relative standard deviation was less than 3·0% in all instances.

The method was tested by applying it to the determination of the analyte in pharmaceuticals. Tablets of Melleril-50 were dissolved in 50 ml of distilled water and the solution was filtered through a paper filter to remove the excipient. Samples with different concentrations of the analyte were prepared, from these solutions and injected into the FIA system; the data obtained were processed as described in the 'Experimental' section. The results obtained showed an agreement with the nominal contents to within ±5%. The samples were stored in topaz flasks, which were kept in the dark in order to avoid the effect of light.

### Conclusion

An open–closed system involving irradiation of the reactor was developed in order to perform kinetic measurements, which were based on the additional development of a photochemical reaction produced by passing the sample plug several times through the circuit. The system can be used with any type of detector and flow-cell, thus avoiding the need to design and construct special flow-cells.

### Acknowledgement

The authors wish to thank the Comisión Interministerial de Ciencia y Tecnología (CICyT) for its financial support (under Grant No. PA86-0146).

### References

1. Birks, J. W., *Chemiluminescence and Photochemical Reaction Detection in Chromatography* (VCH, New York, 1989).
2. Chen, Danhua, Ríos, A., Luque de Castro, M. D. and Valcárcel, M., *Analyst*, **116** (1991), 167–169.
3. Ibid., *Talanta.*
4. León, L. E., Ríos, A., Luque de Castro, M. D. and Valcárcel, M., *Analyst*, **115** (1990), 1549–1552.
5. McKelvie, I. D., Hart, B. T., Cardwell, T. J. and Cattrall, R. W., *Analyst*, **114** (1989), 1459–1463.
6. León, L. E., Ríos, A., Luque de Castro, M. D. and Valcárcel, M., *Analytica Chimica Acta,* **234** (1990), 227–232.
7. Ríos, A., Luque de Castro, M. D. and Valcárcel, M., *Analytical Chemistry,* **57** (1985), 1803–1809.
8. Ibid., *Analytica Chimica Acta,* **179** (1986), 463–468.
9. Ibid., *Journal of Chemical Education,* **June** (1986), 552.
10. Scholten, A. H. M. T., Brinkman, U. A. Th. and Frei, R. W., *Analytica Chimica Acta,* **114** (1980), 137–146.

